# Microbiome Forensic Biobanking: A Step toward Microbial Profiling for Forensic Human Identification

**DOI:** 10.3390/healthcare9101371

**Published:** 2021-10-14

**Authors:** Luciana Caenazzo, Pamela Tozzo

**Affiliations:** Laboratory of Forensic Genetics, Department of Molecular Medicine, University of Padova, 35121 Padova, Italy; luciana.caenazzo@unipd.it

**Keywords:** microbiome, human identification, biobank, forensic

## Abstract

In recent years many studies have highlighted the great potential of microbial analysis in human identification for forensic purposes, with important differences in microbial community composition and function across different people and locations, showing a certain degree of uncertainty. Therefore, further studies are necessary to enable forensic scientists to evaluate the risk of microbial transfer and recovery from various items and to further critically evaluate the suitability of current human DNA recovery protocols for human microbial profiling for identification purposes. While the establishment and development of microbiome research biobanks for clinical applications is already very structured, the development of studies on the applicability of microbiome biobanks for forensic purposes is still in its infancy. The creation of large population microbiome biobanks, specifically dedicated to forensic human identification, could be worthwhile. This could also be useful to increase the practical applications of forensic microbiology for identification purposes, given that this type of evidence is currently absent from most real casework investigations and judicial proceedings in courts.

## 1. Introduction

A biobank is a facility that stores biological material, i.e., tissues and liquids of a living organism—human, animal, plant or microorganism, and collected data, through activities ranging from collection to distribution.

Research biobanks are thus collections of biological material linked with donor data, in particular with clinical and epidemiological data, which can be used for a variety of research projects. The creation of biobanks is closely linked to the development of individualized or personalized medicine [1]. 

The priority objective of personalized medicine is to produce more effective and better adapted therapies for the patient by linking a huge amount of genomic data with individual health data (diseases, therapies, etc.) and other personal data (e.g., on lifestyle, eating habits, physical activity, income, etc.) [2]. This evolution, made possible by developments in genetic analysis and bioinformatics, has for some years been considered a revolution in medicine [3,4]. Public biobanks belong to, or operate on behalf of, public institutions, notably university hospitals, and are financed by the state. In general, they can be distinguished between tissue banks of the pathology units of universities and central hospitals and national biobanks such as those in the United Kingdom [5], Taiwan [6], or Estonia [7]. Public biobanks are not for profit and operate in the spirit of public service for research, while private biobanks are primarily composed of collections of samples and data collected by pharmaceutical companies and clinical research organizations and essentially come from clinical studies. Small biotech and life sciences companies also set up sample collections for specific research projects. There are private biobanks that pursue commercial purposes and provide their samples and data for a fee to researchers or other companies, which offer direct-to-consumer tests and whose residual samples are withheld, subject to participants’ consent, for research purposes [8]. Currently, there are also private–public partnership models in which private investors participate in public biobanks or create and manage biobanks together with public operators [9,10,11].

For research, it is essential that access to samples and data stored in biobanks is simple and open. Indeed, large quantities of samples and data are required in order to obtain statistically relevant results. It is therefore important that biobanks collaborate across borders and provide data in standardized formats so that they can be compared [12]. Over the years, platforms and networks have emerged that provide researchers with information on the collections and data of the various biobanks, facilitate their access, and eventually link collections together [13].

The benefits of a biobank depend entirely on the research projects for which the samples and data are made available. The overall benefit of the donated samples or of the residual part of samples and the respective data corresponds hypothetically to the scientific value and practical importance of all the research projects for which these samples are used. The overall benefit cannot be determined in concrete terms, but only described in a very abstract way by defining the research areas or typology of research projects on which a biobank is focused. This means that potential donors should be informed about the type of research project supported (purpose of the biobank) and about the project selection process, so that they can evaluate the potential usefulness of a donation [14].

## 2. The Challenge of Human Microbiome Research

The study of the human microbiome, the genetic heritage of the bacterial communities present within the human body, has always been considered a difficult task [15]. Microbiota are defined as the population of microorganisms (bacteria, fungi, protozoa, and viruses) that colonize an environment in a given time. The microbiome is the totality of the genetic heritage expressed by the microbiota. Every living organism has its own genome and its own genetic heritage; the microbiome is the genome of the microbiota, the genetic heritage of the whole complex of microorganisms present in the organism [16]. The complex structure in which the microbial communities are organized represents an obstacle to traditional in vitro culture, and the sequencing of the microbiome is problematic due to the enormous amount of data to be managed. However, with the development of recent high-throughput sequencing techniques, remarkable progress has been made in the study of the microbiome. It has thus emerged that the microbiome plays a central role, but this is still to be defined in the state of human health, in its metabolism, and in its interaction with drugs. 

The microbiome is now considered an essential component of the human biological system [17]. Dynamic, plastic, and variable in the various anatomical sites, during the different stages of life, and in relation to endogenous and exogenous factors, it would seem to play a key role even in numerous clinical conditions [18]. Hence there is a need to deepen our knowledge of a world not yet fully explored insofar as the analysis of microbial communities can represent a useful diagnostic and therapeutic tool [19].

The research conducted in this area is based on the pursuit of different objectives, from the study of the composition and functional properties of the microbiome to its complex dynamics and its interaction with the organism that hosts it [20,21]. 

Most of the studies published on the microbiome concern the gut microbiome since it is probably the richest one [22] from a biodiversity standpoint.

The creation of microbiome biobanks can promote research with the purpose of adding a fundamental contribution to the understanding of the relationship between microbiota and diseases [23].

Characterizing and studying the human microbiome means analyzing the genetic material of the microbiota and, to this end, there are two common steps to follow: the first step consists of processing the biological samples and extracting the DNA, while it is later sequenced in order to find the order of the nucleic bases along the DNA chain.

In recent years, there have been rapid advances in molecular sequencing and computational techniques. The spread of application of next-generation sequencing (NGS) or high-throughput sequencing (HTS), has incredibly improved the amount of sequencing data that may also be available for forensic purposes. Using NGS to sequence total DNA obtained from a sample allows the sequencing of the whole genome of a given microorganism and the examination of whole communities of microbes, thus obtaining an overview of the resident microbial population.

The main advantages of next-generation sequencing techniques in order to obtain microbiome profiles from samples and individuals are the high levels of parallelism (hundreds of millions of sequential reads in parallel) and the low costs for the production of the DNA sequences [24]. The identification of the taxonomic membership of the components of microbial communities is also of fundamental importance in the study of the microbiome. For this purpose, comparisons are made between the reads and a database that catalogs the association between a certain genome and a particular taxonomic level.

For decades, microbiology has been almost entirely culture-dependent and early studies of the human microbiome involved the culturing of the microbes. Prior to the advent of NGS technologies, only a limited set of information about the human microbiome was available. Nowadays, there are two approaches that are most frequently used in human microbiome research: amplicon sequencing, which relies on sequencing of taxonomic marker genes (usually 16S ribosomal RNA (rRNA) genes) for bacteria and archaea and metagenomic shotgun sequencing, which simultaneously captures all genetic material, providing sequence information on a randomly picked set of DNA fragments extracted from the sample [25]. The 16S rRNA gene, which encodes the small subunit of the bacterial ribosome, is characterized by species-specific variable regions, which are useful for identifying phylogenetic relationships, and highly similar sequences are grouped into operational taxonomic units (OTUs). The assignment of sequences to OTUs is referred to as binning, performed by one of the following methods: unsupervised clustering of similar sequences, phylogenetic models embedding mutation rates and evolutionary relationships, and supervised methods that assign sequences to taxonomic bins based on labeled training data [26]. The bacterial community can be described in terms of which OTUs are represented, their relative abundance, and/or their phylogenetic relationships. The second culture-independent method is metagenomic shotgun sequencing, which simultaneously captures all the genetic material of a sample, providing sequence information on a randomly selected set of DNA fragments, sequencing all microbial genomes within a sample. The information obtained with this method can be used similarly to a 16S rRNA amplicon sequence to identify which taxa are present and the relative abundance of each, and analyze the functional potential of the microbial community. Using this approach, the possibility of detecting species or strain-specific markers is greatly reduced because the larger the genome(s) characterized, the less read depth would be obtained for any particular site [27].

## 3. The Human Microbiome for Forensic Identification Purposes

While the establishment and development of micobiome research biobanks for clinical applications is already very structured, the development of studies on the applicability of microbiome biobanks for forensic purposes is still in its infancy.

Since the beginning of the 1990s it has been thought that the possibility of typing the genome of bacteria with PCR techniques could be a valid tool to be applied in the forensic field and later, in the 2000s, with the birth of bioterrorism, the applications of forensic microbiology started becoming more established and widespread [28]. An increasing number of scientific contributions have, in the last few years, suggested that in some cases the determination of microbial profiles can be used as forensic evidence or, alternatively, as a complementary element to more traditional forensic evidence [29,30,31]. 

Several studies have suggested that an individual can be identified based on profiles of autochthonous microbes that permanently colonize his/her body [32,33]. However, the level of accuracy in identifying a subject decreases as the number of comparison individuals increases since individuals who share the same environment or the same lifestyles could also share the same microbial patterns [34,35].

Since human identification is a comparative analysis, the microbial trace should be compared with a reference sample constituted of site-related microbial communities in order to be linked to the person who left it behind. In the past few years, several studies have focused on stability in the human microbiome over time [36]. The literature published in this field over the past few years has ascertained clearly enough that microbial communities, although personalized, vary systematically across body sites and time, with intrapersonal differences over time being smaller than interpersonal ones, showing such a high degree of spatial and temporal variability that the degree and nature of this variability can constitute in and of itself an important parameter that is useful in distinguishing individuals from one another [37,38,39,40,41]. It is paramount to make the effort to organically synthesize all results achieved until now and to implement the number of participants to this type of study. Therefore, the observations of the available literature warrant further studies to enable forensic scientists to evaluate the risk of microbial transfer and recovery from various items and to further critically evaluate the suitability of current human DNA recovery protocols for microbial profiling, or establish new protocols to prevent microbial transfer and contamination of forensic evidence [42,43]. 

Therefore, in order to increase knowledge in this fascinating, and relatively new, field of application of forensic microbiology, the creation of large population microbiome biobanks, specifically dedicated to forensic human identification, could be worthwhile. This could also be useful in increasing the practical applications of forensic microbiology for identification purposes, given that this type of evidence is currently absent from most real casework investigations and judicial proceedings in courts.

## 4. Identity and Forensic Identification

Although personhood and identity have never been simple concepts, as we learn more about ourselves as an amalgam of us and the microbes that live on us and within us, we will rethink our concepts of personal identity and normalcy. Our understanding of the human microbiome and its interaction with the human body also has implications for how we conceptualize personal identity. If we consider that each individual’s microbiome is unique, then the microbiome may be incorporated into how we define ourselves as people, and thus it would be important to clarify whether it is possible to identify a person through his/her microbiome. 

The concepts of identification and therefore identity are crucial in forensics, since forensic sciences are commonly aimed at identifying people, toxic substances, and objects. It is abundantly clear that each individual is characterized by physical and biological particularities that distinguish him/her from any other individual and even make him/her unique and unrepeatable within the human species. These differentiations are all the more important the greater the demographic development of the human community of reference, much more so when the community itself becomes a civil society and therefore subject to a legal order. In this case, physical identification corresponds to legal identification, virtually reconstructed, according to two-way reference models relating to the anthropomorphic characteristics and the assignment of personal details, as recognized by the particular legal system that has jurisdiction over the person in question. In the case of Western societies, for example, they would include the name, date of birth, place of birth, citizenship, sex, and some other traits that characterize the individual. If such legal characterizations did not exist, it is easy to understand how any legal relationship and, from a sociological point of view, any type of relationship between individuals would fail, lacking the certainty of the interlocutor’s identifiability. The term identity derives from the medieval term identicus, which in turn is of Latin derivation, with the meaning of “the same”. 

When referring to personal identity, we talk about how to understand and explain how a person can remain the same despite the physical, psychological, existential changes he/she goes through throughout his/her life. The concept of identity in a more scientific sense is made clearer by resorting to the principle of absolute identity, according to which everything is identical to itself and can only be itself. This means that identity is the quality of a thing that makes it the only one and differentiates itself from everything else. At this point, it is normal to ask how it is possible to make a comparison between two individualities if, by definition, the principle of absolute identity prevents us from doing so: the answer is given to us by the principle of relative identity, i.e., the possibility of comparison existing between two terms of comparison which, although being an expression of two distinct individualities (in an absolute sense), can also be considered identical to each other, as ways of being of the same reality.

It is clear that the identity we are now talking about in the forensic field is not to be understood as absolute, but rather relative. In fact, what we are dealing with is nothing other than the comparison between two distinct absolute individualities (think of two genetic profiles to be compared, one of which belongs to a certain person) that are expressions of the same reality [44]. This can be considered the core difference between classification (placing an object in a defined category) and identification (the recognition of uniqueness—that something is one of a kind). Therefore, the term identification refers to the technical-scientific activity aimed at establishing the identity of any material and, in the case of personal identification, the identity of a person. Identity in itself encompasses two concepts: the comparison between two terms and the resulting judgment. No individual can be identified with another; that is, being him/herself and at the same time another subject; it is only itself and is identical to itself only in the instant in which it is observed. An individualization can be viewed as a special case of identification, where the restricted class is populated by only one object. Definitions of individualization in the forensic literature (e.g., fingerprint, footwear marks, or tool marks) systematically refer to the capability of pointing to the right source to the exclusion of all others (objects or persons) [45]. Hence, by default, the size of the population of relevant sources considered at the outset of the examination is systematically set to its maximum, regardless of the specific circumstances of the case. We call this the Earth population paradigm. In that paradigm, the individualization conclusion cannot be reached in a deductive manner, but is de facto probabilistic in nature.

## 5. Human Microbiome Biobanking for Forensic Research in Human Identification

Today, in forensic genetics, human identification is always performed following specific investigative directives which involve observation of the proof, collection, analysis, and scientific interpretation of the results. Once identified, the biological material is collected, stored, and eventually characterized following specific procedures. Then, in the laboratory, DNA is extracted and quantified. Current forensic methods typically rely on targeting genetic markers to create genetic profiles to compare evidentiary items with profiles generated from a reference sample from an individual. With this purpose, specific DNA regions are targeted and amplified by PCR reaction using commercial kits that allow multiplex amplification of specific sets (multiplex) of short tandem repeat (STR) markers, insertion/deletion polymorphisms, or other markers useful for forensic aims. In some cases when the evidentiary sample may be degraded or contain low amounts of DNA (i.e., low-copy number (LCN) DNA), high-copy number (HCN) markers (i.e., the mitochondrial genome or hypervariable regions of the mitochondrial genome) are targeted. 

Other HCN markers, such as skin microbiome genetic markers, may provide additional identifying genetic information that can be used independently or potentially in conjunction with partial human forensic marker profiles [39]. 

Given that human identification is comparative in its nature, when using the microbiome for identification purposes, the microbial profile has to be compared with that of a reference sample of interest in order to be linked to a specific person. In using the microbiome for human identification, it should be considered that the microbiome varies among different tissues and body sites and also over time, although the variability between individuals seems to be greater than the variability between different parts of the same person’s body [42]. This degree of variability constitutes in itself an important element in being able to distinguish the subjects from each other and, therefore, in considering the determination of the microbiome as a useful technique, in association with other more consolidated techniques, for personal identification in the forensic field [46]. 

The application of these new technologies consists of gaining information about the microbiome profile of a wanted person from a trace itself. Its use could be especially useful in investigative cases where there are no potential suspects and no match between the evidence DNA sample under investigation and any genetic profiles entered, for example, in criminal databases [47,48]. Through microbiome prediction starting from biological samples found at the crime scene, probabilistic information may be acquired as to the same characteristics of the sample donor, such as past exposures, visits to other countries, predisposition to certain conditions, sexual practices, diet, and consumption of tobacco, alcohol, and other drugs [49]. The combination of these elements, therefore, narrows the circle of possible perpetrators and facilitates investigations, adding qualitative information that should be integrated with other investigative elements [50]. 

Having tens of thousands of samples stored in dedicated forensic biobanks to be used for forensic studies would allow us to achieve very important objectives. In particular, the first aspect to be addressed is the quantification of diversity of intra-subject species, in order to characterize the microbiome and evaluate its variability in different populations, within the subjects, regardless of their state of health. Secondly, it would be essential to establish the inter-subject variability of the microbiome, quantifying the differences between the bacterial communities of different subjects, considering various environmental factors. Finally, the differences between the microbiome of different subjects, with different lifestyles, could be quantified, in order to evaluate the reliability of the prediction on the lifestyles of those who left a trace starting from the analysis of the microbiome [51].

The creation of this type of “forensic microbiome biobank” would undoubtedly allow for the improvement of technologies for the isolation and analysis of bacterial organisms, the development of a set of sequences of reference microbial genome and the development of new tools for computational analysis and new technologies for sequencing (to be associated with those already in use in many forensic laboratories), which permit the examining of the genome of bacterial communities since the creation of numerous and complex data sets always requires new analysis tools. Above all, given the fact that no standardized technique is available for the purpose of microbiome forensic profiling, it is important to obtain comparable data from different studies. Therefore, the definition of shared objectives, strategies, and protocols in this area represents a precious and irreplaceable opportunity to enrich, compare, and consolidate skills, creating a constantly updated heritage for researchers and forensic scientists [52]. To reach this goal, it is necessary that the institution of biobanks of samples and data also contains reference databases categorized by, for example, type of sample, individual age, race, habits, and ethnicity in order to detect the possibility of determining a “core microbiome” among human subjects. Furthermore, the institutionalization of this type of research within dedicated biobanks may allow for the establishment of one or more centers for the coordination and analysis of data, which manages the processed and unprocessed data, coordinates the analyses and establishes a portal through which it is possible to give international visibility to projects and support international relations. Moreover, thanks to these structures, resources can be widely available and accessible to the scientific community. 

It is also important to have facilities that allow biological material to be stored in the best way to ensure the reproducibility and comparability of research results—above all, because the goal should be to have analytical standards, reference databases, and predictive methods to be applied in concrete cases of judicial investigation. The essential standards are represented by the horizontal requirements for the infrastructure, the competence of the staff of a biobank, the quality management system (QMS), the equipment, the quality control (QC), and the procedures for the management, sample processing, and storage, including method validation and verification.

In the case of microbiome forensic biobanks, samples will be obtained through noninvasive or minimally invasive means, for example, including skin/brushes, oral swabs, saliva samples, nasal swabs, vaginal swabs, and self-collected fecal samples. Moreover, leftover materials collected during endoscopies to collect the gut microbiome may add a minimal additional risk to conducting this type of research [53]. Because the risks of most human microbiome research and biobanks are often negligible, they involve only the lowest measure of “minimal risk” as defined in many regulations. Rhodes et al. propose a new conception and category of risk, that is “de minimis risk”, to appropriately describe the risks in the context of human microbiome research. As they explained, “it entails a degree of risk so low that harms are nominal and unlikely” [54]. However, as we gain a greater understanding of variation in the microbiota that inhabit different parts of the body, as well as the advantages of deep versus minimally invasive sampling, sampling techniques and associated risk–benefit assessments may change. 

The establishment of this type of biobank, due to the very delicate nature of the activities connected to it, should also meet certain regulatory requirements and quality standards that guarantee its correct functioning, impartiality, the presence of all the requirements, and the protection of donors’ personal data. The ethical issues and logistical challenges arising with the use of microbiome biobanks vary with the nature of the research. From a general point of view, we can assume that these are similar to the many concerns raised by other types of biobanks.

The social, ethical, and political concerns and issues pertaining to microbiome identification in forensics are situated within the intersection of civil rights, science, and governance. They are intimately linked to the constitution of new and wider groups of populations as “microbiome suspects”. Such concerns include, but are not limited to, privacy, surveillance, ideological and scientific interpretation of such evidence, and the scientific reliability of microbiome identification, as well as the potential misuse and abuse of criminal investigations. However, the fact that human microbiome research samples also contain human DNA raises concerns about privacy and confidentiality, since these samples can be analyzed in ways that are identifiable. In this respect, we suggest that human microbiome research samples should be treated by biobanks with the same safeguards in terms of privacy and confidentiality as any other human tissue samples or identifying sources of information. 

## 6. Conclusions

The possibility of using the human microbiome as a means of personal identification undoubtedly represents a fascinating and innovative field of research. The establishment of dedicated biobanks for the development of research in this area would allow for the obtaining of reproducible and robust results to allow these techniques to be used in the very near future, even in real criminal cases. Applying new technologies or considering new applications of technologies that have already been consolidated in the forensic and judicial field means proceeding on two tracks: on the one hand, research, to update knowledge; and on the other, to keep in consideration the regulatory and ethical aspects.

## Data Availability

No new data were created or analyzed in this study. Data sharing is not applicable to this article.
